# Identification of established arrhythmogenic right ventricular cardiomyopathy mutation in a patient with the contrasting phenotype of hypertrophic cardiomyopathy

**DOI:** 10.1186/s12881-017-0385-8

**Published:** 2017-03-03

**Authors:** Matthew Neil Bainbridge, Lili Li, Yanli Tan, Benjamin Y. Cheong, Ali J. Marian

**Affiliations:** 1 Human Genome Sequencing Center, Baylor College of Medicine, One Baylor Plaza, Houston, TX 77030 USA; 2 Center for Cardiovascular Genetics, Institute of Molecular Medicine, 6770 Bertner Street, DAC 950H, Houston, TX 77030 USA; 3 Center for Cardiovascular Genetics, Institute of Molecular Medicine, 6770 Bertner Street, DAC 950J, Houston, TX 77030 USA; 4 Department of Radiology, CHI St. Luke’s Health-Baylor St. Luke’s Medical Center, Houston, TX 77030 USA; 5 Center for Cardiovascular Genetics, Institute of Molecular Medicine, University of Texas Health Sciences Center at Houston, and Texas Heart Institute, 6770 Bertner Street, DAC900, Houston, TX 77030 USA

**Keywords:** Cardiomyopathy, Mutation, Plakophilin 2, Precision medicine, Genetics, Case report

## Abstract

**Background:**

Advances in the nucleic acid sequencing technologies have ushered in the era of genetic-based “precision medicine”. Applications of the genetic discoveries to practice of medicine, however, are hindered by phenotypic variability of the genetic variants. The report illustrates extreme pleiotropic phenotypes associated with an established causal mutation for hereditary cardiomyopathy.

**Case presentation:**

We report a 61-year old white female who presented with syncope and echocardiographic and cardiac magnetic resonance (CMR) imaging findings consistent with the diagnosis of hypertrophic cardiomyopathy (HCM). The electrocardiogram, however, showed a QRS pattern resembling an Epsilon wave, a feature of arrhythmogenic right ventricular cardiomyopathy (ARVC). Whole exome sequencing (mean depth of coverage of exons 178X) analysis did not identify a pathogenic variant in the known HCM genes but identified an established causal mutation for ARVC. The mutation involves a canonical splice accepter site (c.2146-1G > C) in the *PKP2* gene, which encodes plakophillin 2. Sanger sequencing confirmed the mutation. *PKP2* is the most common causal gene for ARVC but has not been implicated in HCM. Findings on echocardiography and CMR during the course of 4-year follow up showed septal hypertrophy and a hyperdynamic left ventricle, consistent with the diagnosis of HCM. However, neither baseline nor follow up echocardiography and CMR studies showed evidence of ARVC. The right ventricle was normal in size, thickness, and function and there was no evidence of fibro-fatty infiltration in the myocardium.

**Conclusions:**

The patient carries an established pathogenic mutation for ARVC and a subtle finding of ARVC but exhibits the classic phenotype of HCM, a contrasting phenotype to ARVC. The case illustrates the need for detailed phenotypic characterization for patients with hereditary cardiomyopathies as well as the challenges physicians face in applying the genetic discoveries in practicing genetic-based “precision medicine”.

**Electronic supplementary material:**

The online version of this article (doi:10.1186/s12881-017-0385-8) contains supplementary material, which is available to authorized users.

## Backgrounds

Technological advances in nucleic acid sequencing have enabled identification of the genetic variants (GVs) across the genome and have ushered in utilization of the GVs in practice of medicine. Genetic-based “personalized medicine” or “precision medicine” advocates for exploiting the information content of the GVs to determine susceptibility to disease, individualize therapy, in order to maximize gain and to reduce the side effects, prognosticate, and assess the clinical outcomes. In a broader definition, “precision medicine” promotes utilizing various individualized indicators, encompassing metabolomics, transcriptomics, genomics, proteomics, environmental exposures, and microbiome, among others, to tailor the medical management of an individual.

Single gene disorders, whereby the effect size of the GVs are relatively large, are the prototypic diseases for the applications of genetic-based “precision medicine”. However, there is considerable phenotypic variability and pleiotropic effects, even in single gene disorders. The variability typically results from different mutations in a given causal gene causing distinct phenotypes. The point has been illustrated for hereditary cardiomyopathies, whereby different mutations in genes encoding sarcomere proteins can cause either hypertrophic cardiomyopathy (HCM) or dilated cardiomyopathy (DCM) and affect severity of the phenotypic expression [1–3]. However, phenotypic variability of a specific mutation associated with two distinct cardiomyopathy phenotypes is less well appreciated. We report an interesting patient who exhibits the classic phenotype of hypertrophic cardiomyopathy (HCM) but carries a rare mutation that has been established to cause the contrasting phenotype of arrhythmogenic right ventricular cardiomyopathy (ARVC).

## Case presentation

**Fig. 1:**
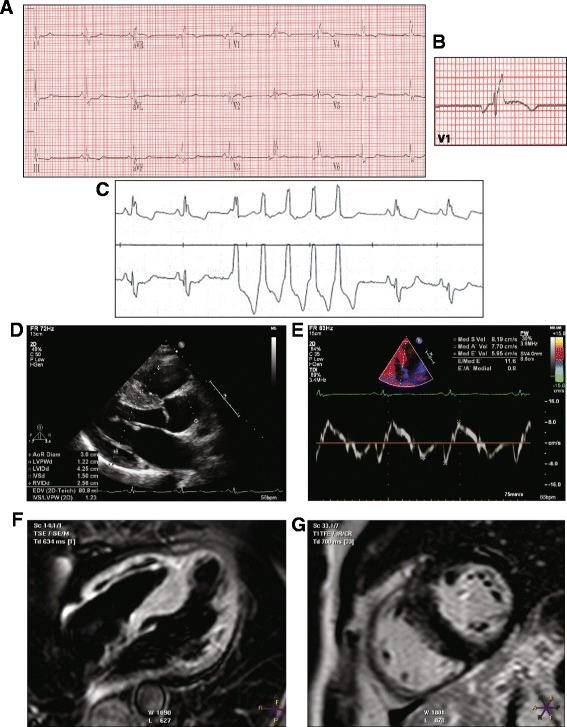
Phenotypic data. **a** 12-lead electrocardiogram is notable for a QRS morphology resembling right bundle branch block with an epsilon wave, a pattern similar to that observed in Brugada syndrome. **b** QRS morphology in lead V1 showing an Epsilon wave. **c** A rhythm strip from a Holter monitoring recording showing a 5-beat non-sustained ventricular tachycardia. **d** A parasternal long axis echocardiographic view showing interventricular septal hypertrophy. **e** Septal tissue Doppler imaging, showing reduced velocities. **f** and **g**. CMR images, fat saturation sequence **f** and cardiac function **g**, showed a normal RV size and function and no gadolinium enhancement in the RV

The patient is a 61-year white (European decent, non-Finish) female who presented with unexplained syncope 4 years ago. She had no prior history of syncope, cardiac arrhythmias, cardiovascular diseases, and no family history of cardiomyopathies. She had a past medical history of depression and mild hypothyroidism, which have been controlled with medical therapy. Her physical examination was unremarkable. A 12-lead ECG was notable for possible left atrial enlargement, q waves in L1 and aVL, a QRS morphology resembling right bundle branch block with an epsilon wave, and non-specific ST and T changes (Fig. 1a and b). Holter monitoring (24 h) showed a run of 5-beat ventricular tachycardia and frequent ventricular premature beats and couplets (Fig. 1c). An echocardiogram showed asymmetric left ventricular hypertrophy with an interventricular septal thickness of 19 mm (normal range: 6–11 mm), a posterior wall thickness of 1.1 cm, a hyperdynamic left ventricle with an ejection fraction of > 60%, a normal right ventricular size and function, and reduced myocardial tissue Doppler velocities (Fig. 1, d–g). She had no overload conditions, such as systemic arterial hypertension or valvular pathology to explain cardiac hypertrophy. The findings were consistent with the diagnosis of HCM. She was treated with a beta-blocker for HCM, which she soon discontinued. She was taking aspirin (81 mg/d), cholecalciferol (1000 units/d), and levothyroxine (75 mg/d), and received monthly intramuscular injection of paliperidone palmitate for depression.

**Fig. 2:**
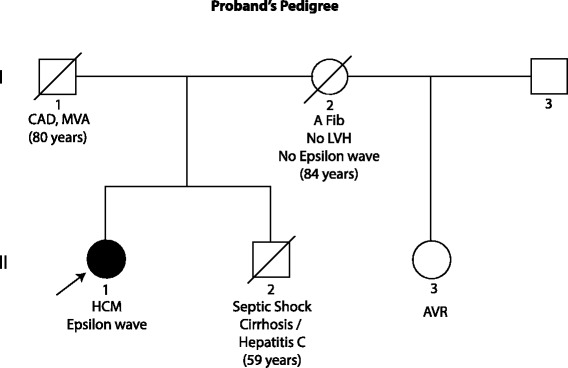
Pedigree. Pedigree of the proband containing the phenotypic data of the key members is shown. *Square box* and *circle* represent male and female members respectively. *Full circle* indicates an affected member. The / symbol indicates a deceased individual. Age at the time of death is shown. Abbreviations: CAD: Coronary artery disease; MVA: Motor vehicle accident; A Fib: Atrial fibrillation; LVH: left ventricular hypertrophy; HCM: Hypertrophic cardiomyopathy; AVR: Aortic valve replacement. Age at the time of death is shown in parenthesis are shown in the figure

The proband had no family history of HCM or ARVC (Fig. 2). She was never married and had no child. Proband’s mother (I-2 in the Pedigree) had atrial fibrillation with a controlled ventricular rate at 80 bpm and left anterior fascicular block on a 12-lead ECG but no Epsilon wave or evidence of cardiac hypertrophy. Individual I-2 had an echocardiogram at 80 years of age, which was 4 years prior to her death, which showed a normal left ventricular size and function with an interventricular wall thickness of 0.9 cm. She had no evidence of right ventricular dilatation or dysfunction on the echocardiogram. Proband’s father (I-1 in the Pedigree) had a history of coronary artery disease and died in a car accident at an old age. Proband’s brother (II-2) had no history of heart disease and died from septic shock and respiratory failure, as complications of liver cirrhosis caused by hepatitis C. Proband’s half-sister had degenerative aortic valve disease and underwent aortic valve replacement in her 50s.

Whole exome sequencing (WES) was performed on the Illumina 2500 platform as part of clinical genetic testing by a commercial company. Sequencing produced 2 × 100 bp reads, which were aligned to the human reference genome (hg19) using BWA-Mem (v0.7.8) [4]. The BAM files, containing the sequence alignment data, were analyzed to identify the GVs using Platypus (v0.8.1) [5]. CASSANDRA (v2.0) was used to annotate the GVs [6]. The discovered variants were filtered by minor allele frequency <1% (ExAc overall minor allele frequency, http://exac.broadinstitute.org/) and prioritized providing they met any of the following criteria:

1. Stop gain, splicing (2 bp of canonical site), and any insertion or deletion
2. Pathogenic or likely pathogenic as annotated by ClinVar [7]
3. Nonsynonymous variants that met all the following criteria
  - CADD_PH (combined annotation-dependent depletion phred score) score > 10
  - Medium or high likelihood of being deleterious as assessed by mutation assessor [8],
  - PolyPhen2:HVAR [9] score > 0.9

The mean depth of coverage of exons in the entire dataset was 178 times. In the entire data set, 95.3% of the exons had at least 10 sequence reads. Further analysis of the 15 genes known to cause HCM and phenocopy conditions, namely *MYBPC3*, *MYH7*, *TNNT2*, *TNNI3*, *TPM1*, *ACTC1*, *MYL2*, *MYL3*, *FH11*, *NEXN*, *PLN*, *GLA*, *LAMP2*, and *PRKAG2* showed 100% had at least 10 reads and 98% had at least 20 reads (Table 1).

**Table 1:** Coverage of known HCM and HCM-phenocopy genes in the WES data

Gene	Proportion of coding region covered at given level		
	1x	10x	20x
*MYBPC3*	1	1	0.9663
*MYH7*	1	1	1
*TNNT2*	1	1	0.9989
*TNNI3*	1	1	0.8674
*TPM1*	1	0.9993	0.9289
*ACTC1*	1	1	1
*MYL2*	1	1	1
*MYL3*	1	1	1
*FH11*	1	1	1
*NEXN*	1	1	1
*PLN*	1	0.9983	0.9883
*GLA*	1	1	1
*LAMP2*	1	1	1
*PRKAG2*	1	1	0.9589

A total of 80 candidate GVs in the WES data, including 29 insertions/deletions, 16 variants a introducing premature stop codon, 3 variants affecting the splicing sites, and 32 deleterious non-synonymous variants met the filtering criteria (Additional file 1: Table S1). No pathogenic variant in any of the established causal genes for HCM was identified. Given the high read coverage (Table 1), the possibility of missing a causal variant in HCM genes was very low. Ten of the candidate pathogenic variants were in genes associated with a phenotype per Online Mendelian Inheritance in Man (OMIM) database [10]. The list included *CLDN16*, *COL6A2*, *COL6A3*, *FANCD2*, *GPR179*, *LFNG*, *NOTCH3*, *PKP2*, *POF1B*, *TGIF1* (Table 2).

**Table 2:** Variants identified in the WES data whose corresponding genes have been associated with mendelian disorders

Gene containing a pathogenic variant	Disease/Phenotype listed in OMIM	Phenotype in the proband
*CLDN16*	• Hypomagnesemia	Not documented
*COL6A2*	• Bethlem myopathy 1 • Ullrich congenital muscular dystrophy 1 • ?Myosclerosis	Not documented
*COL6A3*	• Bethlem myopathy 1 • Dystonia 27 • Ullrich congenital muscular dystrophy 1	Not documented
*FANCD2*	• Fanconi anemia	Not documented
*GPR179*	• Night blindness, Congenital stationary (complete) • 1E, autosomal recessive	Not documented
*LFNG*	• ?Spondylocostal dysostosis 3\autosomal recessive	Not documented
*NOTCH3*	• Cerebral arteriopathy with subcortical infarcts and leukoencephalopathy 1 • Lateral meningocele syndrome • ?Myofibromatosis, infantile 2	Not documented
*PKP2*	• Arrhythmogenic right ventricular cardiomyopathy	Suggestive
*POF1B*	• Premature ovarian failure 2B	Not documented
*TGIF1*	• Holoprosencephaly 4	Not documented

**Fig. 3:**
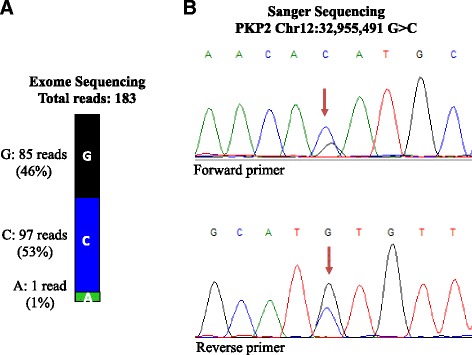
The number of reads in WES and confirmation of the genetic variant by Sanger sequencing. **a** Whole exome sequencing data showing the number of reads covering the c.2146-1G > C variant in the *PKP2* gene. **b** Sanger sequencing showing heterozygous G > C read out, confirming the WES data

Among the pathogenic variants identified, the most plausible pathogenic variant for the phenotype in the proband is a rare splice variant (c.2146-1G > C) in the *PKP2* gene (NM_001005242.2), which encodes plakophilin 2 (<1 in 20,000 in ExAc database in European – Non-Finnish population). *PKP2* is the most common causal gene for ARVC [11]. The variant is predicted to disrupt the canonical splice acceptor site in exon 10 of the *PKP2* gene and has been reported in multiple independent probands/families to cause ARVC [11–14]. The variant was read 183 times in the WES data and was confirmed by Sanger sequencing (Fig. 3). Because the c.2146-1G > C variant is known to cause ARVC [11–14], the patient was further evaluated for this distinct form of cardiomyopathy. The ECG and echocardiogram were unchanged. CMR showed interventricular septal hypertrophy but no evidence of right ventricular dilatation, dysfunction, aneurysm, or fibro-adipocyte infiltration (Fig. 1, panels f and g, and Additional file 2: Movie S1 and Additional file 3: Movie S2).

## Discussion

Although different mutations in a single gene can cause distinct phenotypes, the observed association of a single mutation with two distinct and contrasting phenotypes, namely HCM and ARVC, is rare, if not unique. The former is characterized by cardiac myocyte hypertrophy, classically in the left ventricle and the latter by myocyte atrophy, apoptosis, and excess fibro-adipocytes, classically in the right ventricle. The patient had a clear clinical diagnosis of HCM, as evidenced by the presence of asymmetric cardiac hypertrophy with a predominant involvement of the interventricular septum, on multiple echocardiograms and CMR, in the absence of a secondary cause, such as systemic arterial hypertension or aortic stenosis. However, the patient did not meet the clinical criteria for the diagnosis of ARVC, despite carrying a well-established mutation in the most common causal gene for ARVC [11]. The QRS pattern resembling an epsilon wave is considered a major, albeit insufficient, diagnostic criterion for the ARVC, evoking possible ARVC [15]. The absence of a full ARVC phenotype in the proband might reflect incomplete penetrance, which is a known feature of the *PKP2* variants [14]. The Epsilon wave observed in the proband is not a feature of HCM but is a major diagnostic criterion for ARVC. The electrocardiographic pattern also resembles the pattern observed in Brugada syndrome [16], which is also associated with the *PKP2* mutations [17]. However, cardiac hypertrophy is not a feature of the Brugada syndrome.

The published evidence for the pathogenic role of the c.2146-1G > C variant in ARVC is strong [11–13]. However, its causal role in HCM is uncertain. Rare variants, including the pathogenic variants, are population-specific. Consequently, detection of a pathogenic variant, previously identified as a disease-causing variant, in a single individual is insufficient to imply causality. Indeed, unambiguous ascertainment of genetic causality, regardless of the causal gene and variant, is exceedingly challenging if not impossible. HCM in this patient might be caused by undetected pathogenic variants, structural variants, and variants did not meet the filtering criteria [18]. The exome of this patient also contained a rare variant in the *OBSCN* gene, which is a candidate gene for DCM but not an established causal gene for HCM [19]. As shown in Table 1, WES had provided an excellent coverage to 15 known HCM and HCM-phenocopy genes. Thus, it is very unlikely that a pathogenic variant in the common HCM gene was undetected. Finally, it is also possible that a copy number variant or a large deletion, not detected by whole exome sequencing, is responsible for HCM in this case, albeit such variants are found in < 1% of HCM cases [20]. Thus, one cannot exclude concomitant presence of HCM and ARVC phenotypes in this case.

## Conclusions

The case illustrated extreme phenotypic pleiotropy associated with a *PKP2* mutation, which is an established causal mutation for ARVC and yet this pathogenic variant was found in a patient with the well-defined contrasting phenotype of HCM. The diversity of the phenotypic expression of GVs does not diminish the clinical utilities of genetic testing in hereditary cardiomyopathies. To the contrary, the findings emphasize the need for better understanding of the phenotypic variability associated with the GVs by placing the focus on detailed phenotypic and genetic characterization of the patient. The ambiguities in genotype-phenotype correlation in this case illustrate the challenges physicians face in applying the genetic discoveries to the practice medicine. To quote Sir William Osler, the practice of “*medicine is a science of uncertainty and an art of probability*”. It seems to remains so, even in the era of “precision medicine”.

## Additional files

Additional file 1: Table S1. List of candidate pathogenic variants in proband’s exome met the filtering criteria; Description: 80 pathogenic variants are listed. (PDF 75 kb)
Additional file 2: Movie S1. Cine CMR 1; Description: CMR showing normal RV and LV function and no RV dilatation. (MOV 1995 kb)
Additional file 3: Movie S2. Cine CMR 2; Description: CMR showing normal RV and LV size and no RV wall thinning or aneurysm. (MOV 2605 kb)

## Abbreviations

ARVC: Arrhythmogenic cardiomyopathy
CMR: Cardiac magnetic resonance imaging
DCM: Dilated cardiomyopathy
GVs: Genetic variants
HCM: Hypertrophic cardiomyopathy
PKP2: Plakophilin 2
WES: Whole exome sequencing
