# Supplementary material for: Identification of established arrhythmogenic right ventricular cardiomyopathy mutation in a patient with the contrasting phenotype of hypertrophic cardiomyopathy
Source: BMC Med Genet. 2017 Mar 3;18:24. doi: 10.1186/s12881-017-0385-8 (PMC5335712; doi:10.1186/s12881-017-0385-8)
Supplement: Additional file 1: Table S1. — List of candidate pathogenic variants in proband’s exome met the filtering criteria; Description: 80 pathogenic variants are listed. (PDF 75 kb) [file 12881_2017_385_MOESM1_ESM.pdf]

Table S1

Gene	Chrom	Position (hg19)	Reference	Variant	Variant Type	Variant effect	EXAC Allele Frequency
AGAP6	10	51768674	CAA	C	frameshift deletion	NM_001077665:exon8:c.790_791del:p.264_264del	0
CELA1	12	51740414	GAC	G	frameshift deletion	NM_001971:exon1:c.7_8del:p.3_3del	0
CLDN16	3	190106071	AG	A	frameshift deletion	NM_006580:exon1:c.164delG:p.R55fs	0
CLSTN2	3	140251317	CT	C	frameshift deletion	NM_022131:exon9:c.1497delT:p.A499fs	0
CTU2	16	88780634	AGGTGTG	A	frameshift deletion	NM_001012759:exon10:c.1097_1097del:p.366_366del	0
DCHS2	4	155244401	TTTTG	T	frameshift deletion	NM_001142552:exon8:c.4095_4098del:p.1365_1366del	0
FANCD2	3	10088406	TAGTA	T	frameshift deletion	NM_001018115:exon15:c.1278_1278del:p.426_426del	0
GPRC5D	12	13102481	AGGCATTGCCTTGTAAAG	A	frameshift deletion	NM_018654:exon1:c.821_837del:p.274_279del	3.45E-03
HDGFRP2	19	4499632	AAGCTGGCCGGGGAGG	A	frameshift deletion	NM_001001520:exon14:c.1721_1722del:p.574_574del	0
LFNG	7	2552881	AGATG	A	frameshift deletion	NM_001166355:exon2:c.139_142del:p.47_48del	0
NOC3L	10	96110026	CA	C	frameshift deletion	NM_022451:exon9:c.971delT:p.L324fs	8.24E-04
OR10Q1	11	57995644	GA	G	frameshift deletion	NM_001004471:exon1:c.703delT:p.S235fs	4.37E-04
OR13C2	9	107367391	ATGTT	A	frameshift deletion	NM_001004481:exon1:c.514_517del:p.172_173del	0
OR5H6	3	97983437	TA	T	frameshift deletion	NM_001005479:exon1:c.310delA:p.K104fs	0
POLR3C	1	145592761	TG	T	frameshift deletion	NM_006468:exon15:c.1533delC:p.A511fs	8.65E-04
PRSS3	9	33797930	GAC	G	frameshift deletion	NM_007343:exon3:c.476_477del:p.159_159del	0
TGIF1	18	3452222	CT	C	frameshift deletion	NM_170695:exon1:c.246delT:p.P82fs	0
TSSC1	2	3358354	CA	C	frameshift deletion	NM_003310:exon2:c.92delT:p.L31fs	0
ZC3H8	2	112988507	CTTCTT	C	frameshift deletion	NM_032494:exon8:c.859_863del:p.287_288del	1.98E-03
ZFPM1	16	88599700	CTCTG	C	frameshift deletion	NM_153813:exon10:c.1335_1338del:p.445_446del	0
ZNF142	2	219507409	TTGACA	T	frameshift deletion	NM_001105537:exon8:c.3825_3829del:p.1275_1277del	0

Table S1

C20orf197	20	58645791	A	AG	frameshift insertion	NM_173644:exon4:c.210dupG:p.K70fs	2.97E-04
CDCP2	1	54605318	T	TG	frameshift insertion	NM_201546:exon4:c.1224dupC:p.M409fs	0
HRNR	1	152185812	C	CAG	frameshift insertion	NM_001009931:exon3:c.8292_8293insCT:p.G2765fs	0
KRTAP9-1	17	39346626	G	GTGGGT	frameshift insertion	NM_001190460:exon1:c.488_489insTGGGT:p.C163fs	0
PRUNE2	9	79324134	GA	GGTGACTGTTGCAG	frameshift insertion	NM_015225:exon8:c.3055_3056insCTGCAACAGTCAC:p.S1019fs	0
PRUNE2	9	79324153	G	GTTGC	frameshift insertion	NM_015225:exon8:c.3036_3037insGCAA:p.Q1013fs	1.66E-05
PTCHD2	1	11579472	T	TC	frameshift insertion	NM_020780:exon8:c.1951dupC:p.A650fs	8.27E-06
PTCHD3	10	27702256	G	GC	frameshift insertion	NM_001034842:exon1:c.923dupG:p.G308fs	0
ADAMTSL5	19	1506031	G	A	nonsynonymous	NM_213604:exon12:c.C1399T:p.R467W	4.67E-03
APC2	19	1457002	G	C	nonsynonymous	NM_005883:exon9:c.G967C:p.G323R	0
ATP8B4	15	50223353	C	A	nonsynonymous	NM_024837:exon16:c.G1605T:p.L535F	1.72E-03
AUTS2	7	69064650	C	T	nonsynonymous	NM_001127232:exon1:c.C11T:p.P4L	7.22E-04
BHLHB9	X	102004875	T	C	nonsynonymous	NM_001142524:exon4:c.T952C:p.C318R	4.78E-03
BMP8B	1	40254155	C	A	nonsynonymous	NM_001720:exon1:c.G3T:p.M1I	0
BTNL3	5	180416029	A	G	nonsynonymous	NM_197975:exon1:c.A1G:p.M1V	0
C7orf72	7	50175760	T	G	nonsynonymous	NM_001161834:exon5:c.T934G:p.F312V	2.15E-03
CALHM1	10	105218160	C	T	nonsynonymous	NM_001001412:exon1:c.G349A:p.V117I	1.11E-03
CMYA5	5	79024833	G	A	nonsynonymous	NM_153610:exon2:c.G245A:p.G82E	3.06E-04
COL6A2	21	47542052	C	T	nonsynonymous	NM_058174:exon19:c.C1552T:p.P518S	8.79E-03
COL6A3	2	238277379	C	T	nonsynonymous	NM_057166:exon7:c.G2906A:p.R969Q	0.01
DNAJC5G	2	27499675	G	A	nonsynonymous	NM_173650:exon3:c.G79A:p.G27S	0
DOK3	5	176931030	A	T	nonsynonymous	NM_024872:exon6:c.T1445A:p.L482Q	0
GPR179	17	36486848	C	G	nonsynonymous	NM_001004334:exon11:c.G2604C:p.E868D	5.06E-03
HIPK1	1	114514635	G	C	nonsynonymous	NM_152696:exon15:c.G3203C:p.R1068T	4.12E-05

Table S1

KIR3DL2	19	55378166	G	A	nonsynonymous	NM_006737:exon9:c.G1348A:p.G450S	2.30E-03
MGA	15	41989088	G	A	nonsynonymous	NM_001080541:exon3:c.G1880A:p.R627Q	0
MYLK4	6	2678541	C	T	nonsynonymous	NM_001012418:exon10:c.G953A:p.C318Y	1.85E-03
NOTCH3	19	15276238	T	C	nonsynonymous	NM_000435:exon31:c.A5756G:p.E1919G	0
OSGIN1	16	83992895	A	G	nonsynonymous	NM_182981:exon3:c.A98G:p.Y33C	1.52E-03
PIGC	1	172410967	G	A	nonsynonymous	NM_153747:exon2:c.C796T:p.P266S	0
POF1B	X	84563220	C	T	nonsynonymous	NM_024921:exon10:c.G960A:p.M320I	9.07E-05
RGS22	8	100994172	T	C	nonsynonymous	NM_001286692:exon22:c.A3317G:p.E1106G	3.72E-03
SLC3A2	11	62649421	G	T	nonsynonymous	NM_001013251:exon2:c.G481T:p.G161C	8.48E-04
SLCO5A1	8	70667793	C	T	nonsynonymous	NM_030958:exon4:c.G1124A:p.R375Q	4.17E-03
STXBP5L	3	121126425	G	T	nonsynonymous	NM_014980:exon24:c.G2995T:p.A999S	9.43E-04
TRAPPC10	21	45483598	C	T	nonsynonymous	NM_003274:exon7:c.C970T:p.P324S	1.98E-04
UBASH3A	21	43836715	T	A	nonsynonymous	NM_001001895:exon5:c.T718A:p.Y240N	6.60E-05
ZC3H13	13	46541962	C	T	nonsynonymous	NM_015070:exon15:c.G3998A:p.R1333H	0
ZDHHC14	6	158094126	G	A	nonsynonymous	NM_024630:exon9:c.G1439A:p.R480H	2.51E-05
ZNF318	6	43307399	G	A	nonsynonymous	NM_014345:exon10:c.C4337T:p.P1446L	6.33E-03
PIWIL3	22	25124143	C	T	splicing	NM_001255975:exon16:c.1905+1G>A	5.35E-03
PKP2	12	32955491	C	G	splicing	NM_004572:exon12:c.2146-1G>C	4.94E-05
SLC24A2	9	19550134	C	G	splicing	NM_020344:exon8:c.1479+1G>C	0
CABLES1	18	20715874	G	T	stopgain	NM_001100619:exon1:c.G148T:p.E50X	0
CCDC66	3	56591278	T	TGGGGTAAGCA	stopgain	NM_001141947:exon1:c.8_9insGGGGTAAGCA:p.L3_G4delinsLGX	0
CEP104	1	3761541	G	A	stopgain	NM_014704:exon6:c.C496T:p.R166X	2.47E-05
FUT1	19	49253809	G	A	stopgain	NM_000148:exon4:c.C730T:p.Q244X	0
GLP2R	17	9745887	G	A	stopgain	NM_004246:exon4:c.G458A:p.W153X	0

Table S1

HS3ST6	16	1962046	CG	TA	stopgain	NM_001009606:exon2:c.480delC:p.Y160X	0
LGALS8	1	236702209	CA	TG	stopgain	NM_201543:exon5:c.166delA:p.M56X	0
OBSCN	1	228550291	C	T	stopgain	NM_001271223:exon91:c.C21547T:p.R7183X	0
OR2T35	1	248801602	T	TCA	stopgain	NM_001001827:exon1:c.957_958insTG:p.I320_R321delinsX	0
PITRM1	10	3208567	T	TGCACGCTAGGGAAGAGAGAGGAATG	stopgain	NM_001242309:exon3:c.175_176insCATTCCTCTCTCTTCCCTAGCGTGC:p.Q59_F60delinsPFLSLPX	0
PKHD1L1	8	110477066	C	T	stopgain	NM_177531:exon49:c.C8005T:p.Q2669X	1.33E-03
PREX2	8	68993013	AT	GC	stopgain	NM_025170:exon17:c.1819delT:p.L607X	0
PSG11	19	43529026	CA	TG	stopgain	NM_002785:exon2:c.246delT:p.Y82X	0
STAP2	19	4328755	G	C	stopgain	NM_017720:exon6:c.C507G:p.Y169X	0
TAS2R31	12	11183035	C	T	stopgain	NM_176885:exon1:c.G900A:p.W300X	9.53E-03
ZNF729	19	22498942	GT	CC	stopgain	NM_001242680:exon4:c.2724delT:p.C908X	0
